# State of the evidence on economic impacts of smoke-free policies in the tourism sector: A narrative literature review

**DOI:** 10.18332/tid/211072

**Published:** 2025-12-04

**Authors:** Qinghua Nian, Ryan D. Kennedy, Kerstin Schotte, Hebe Gouda, Emily Xing, Saana Kataria, Kevin Welding

**Affiliations:** 1 Department of Health, Behavior and Society, Institute for Global Tobacco Control, Johns Hopkins Bloomberg School of Public Health, Johns Hopkins University, Baltimore, United States; 2 Department of Health Promotion, No Tobacco Unit, World Health Organization, Geneva, Switzerland

**Keywords:** smoke-free policy, tourism, economic impact, hospitality, casino

## Abstract

Article 8 of the World Health Organization Framework Convention on Tobacco Control (WHO FCTC) obligates Parties to enact policies that create 100% smoke-free environments in enclosed workplaces, public places and public transport. This narrative literature review examines studies reporting economic impact of smoke-free policies on tourism industry sectors including hotels, casinos/gambling venues, and sporting venues. A literature search was conducted across academic and gray literature published between 1 January 2004 and 18 June 2024, using the Scopus, Embase, and JSTOR databases. Search terms included variations of ‘smoke-free’ and ‘tourism’, ‘hospitality’, ‘casino’, ‘hotel’, and other related terms. Studies were included if they reported direct or indirect economic impacts of smoke-free policies on the tourism sector. The screening process involved an initial review of titles and abstracts, followed by full-text assessment for eligibility. Database searching identified 692 articles, of which 37 met the inclusion criteria. Nearly all identified studies (95%) focused on economic impacts in high income countries. The majority (76%) reported neutral or positive economic impacts following the implementation of smoke-free policies. There is evidence that most hotels and other hospitality venues experienced stable or improved revenues, increased customer satisfaction, and enhanced employee health outcomes after going smoke-free. Some evidence indicates that certain casinos experienced short-term revenue declines. Comprehensive smoke-free policies were more consistently associated with economic benefits, while partial policies often produced mixed results, commonly attributed to enforcement challenges. This review supports the evidence that comprehensive smoke-free policies aligned with WHO FCTC Article 8 deliver both health and economic benefits without harming the tourism sector. Findings can help policymakers counter tobacco industry claims and build political support for stronger smoke-free policies, especially in tourism-dependent jurisdictions. The lack of studies from low- and middle-income countries highlights the need for further research in these contexts.

## INTRODUCTION

Tobacco use remains a leading cause of death globally, responsible for 7.7 million deaths annually, including 1.2 million due to secondhand smoke (SHS) exposure^1,2^. SHS poses significant health risks, contributing to severe conditions such as cardiovascular disease and cancer^3^. To mitigate these risks, comprehensive smoke-free laws have been implemented as a critical public health measure. These laws have been shown to significantly reduce SHS exposure and contribute to declines in youth smoking rates^4,5^.

Article 8 of the World Health Organization Framework Convention on Tobacco Control (WHO FCTC) mandates that Parties adopt and enforce comprehensive smoke-free laws, which prohibit smoking in all indoor workplaces, public transport, and public spaces, without exemptions for hospitality venues such as bars, restaurants, and casinos^6^. As of 2022, 74 countries had enacted comprehensive smoke-free policies at healthcare facilities, educational facilities, universities, government facilities, indoor offices and workplaces, restaurants, pubs and bars, and public transport, protecting an estimated 2.1 billion people^7^. Smoke-free policies can be implemented at various levels, including the business, local, and national levels. For example, some hotels have adopted smoke-free policies^8^. Regarding hotels, 68 countries have implemented smoke-free policies covering both main areas and guest rooms^7^. However, 128 jurisdictions, including high-, middle-, and low-income countries, still lack comprehensive smoke-free policies, leaving large portions of the global population exposed to SHS^9^.

Beyond providing health benefits, smoke-free policies offer significant economic advantages. Globally, smoking-related costs, including healthcare expenditures and productivity losses, are estimated to total $1.4 trillion annually, representing 1.8% of global GDP in 2021^10^. Making workplaces and public spaces smoke-free can lessen these financial burdens by reducing healthcare costs, boosting productivity, and lowering insurance premiums^11^. However, despite these advantages, debates persist regarding the economic impact of smoke-free environments on tourism, especially in regions where tourism is a vital economic driver^12^. Allwright^13^ examined the impact of a smoking ban on hospitality sector employees (not limited to those in tourist-focused settings) in 2008 and suggested that it resulted in cost savings due to a healthier work environment, reduced absenteeism, lowered fire risks, and decreased building maintenance costs. Several large hotel chains adopted 100% smoke-free policies across all their properties to protect employee health and enhance guest comfort, leading other businesses to follow their example^8^. However, these voluntary policies adopted by some companies do not create smoke-free environments across the hospitality sector. In the absence of comprehensive smoke-free policies, product offerings can emerge that permit smoking to take advantage of differing practices. Research examining Canadian Airbnb listings found that venues that allowed smoking were on average less expensive than smoke-free venues, underscoring the role of smoke-free policies in leveling the economic playing field within the hospitality sector^14^.

Evidence widely suggests that the implementation of comprehensive smoke-free policies does not have adverse economic consequences for restaurants, bars, and cafes. Scollo et al.^15^ reviewed studies that examined the economic impact of smoke-free laws on restaurants and bars. This review reported that rigorous studies consistently reported neutral or positive effects on business revenues following the implementation of smoke-free policies. To date, there has not been an effort to synthesize our understanding of how smoke-free policies may impact other tourism sectors, such as lodging (which includes hotels, motels, hostels, vacation rentals, etc.), resorts, casinos/ gambling, sports, cruising, and beaches. This literature review aims to address that gap by examining both academic and gray literature to assess the economic consequences – whether neutral, positive, negative, or mixed – of smoke-free policies in tourism sectors beyond restaurants and bars. By doing so, this review seeks to provide a comprehensive understanding of how such policies impact the wider tourism economy and identify areas where further research is needed.

The present study employed a narrative literature review, an approach suitable given its broad, flexible focus and the inclusion of a variety of studies. The initial sample of literature included research articles, reviews, conference papers, letters, editorials, book chapters, and books published between 1 January 2004 and 18 June 2024. The start date was chosen because Ireland, the first country to enact a national smoke-free policy, implemented its policy in 2004. We searched Scopus, Embase and JSTOR databases. Search terms included combinations and variations of: [smoke-free OR smokefree] AND [tourism OR hospitality OR beach* OR cruise* OR casino OR hotel OR resort OR spa]. We used Covidence, a web-based collaboration platform, to manage the identified citations.

Articles were included in the study if they contained content about economic impacts on tourism related to a smoke-free policy, including direct impacts on profits, revenues, visits, or similar, and/or indirect impacts including changes in healthcare costs, employee productivity, employee absenteeism, hotel insurance premiums, maintenance costs including cleaning, guest satisfaction, and worker safety. Articles were included if the direct or indirect impacts included the tourism sector including hotels/accommodation, resorts, casinos, spas, golf courses/sporting, theatre, festivals, museums, cruises and similar. If a study only reported the economic impacts of a smoke-free policy on restaurants/cafes/bars, the study was excluded. Articles that only reported economic impacts to the tobacco industry, or were authored and/or funded by the tobacco industry or published in an industry journal, were also excluded.

Identified articles were reviewed following the PRISMA process, including a title/abstract review, followed by a full text review and content classifications. One coder screened each title and abstract. Two coders independently reviewed the full text of all reports retrieved. Discrepancies were resolved through discussion and consensus. Classifications and themes that were identified *a priori* include where the study took place, characteristics of the smoke-free policy (comprehensive or not), types of hospitality environments studied, and what economic impacts were reported.

The articles included in this narrative review were examined and classified according to their respective WHO Regional Offices: Regional Office for Africa (AFRO), Regional Office for the Americas (AMRO), Regional Office for the Eastern Mediterranean (EMRO), Regional Office for Europe (EURO), Regional Office for South-East Asia (SEARO), and Regional Office for the Western Pacific (WPRO)^16^. Additionally, countries and jurisdictions were classified following the World Bank’s income classifications^17^. For the purposes of this review, countries were grouped into three categories: low-and middle-income countries (LMIC), high-income countries (HIC), or studies from both LMIC and HIC.

Each article was also classified based on thematic content. *A priori* codes were developed for two key smoke-free policy themes: comprehensive smoke-free policy and partial smoke-free policy. These codes were defined as follows:

- Comprehensive Smoke-Free Policy: Defined as policies that prohibit smoking in all indoor workplaces, public spaces, and public transportation without exceptions, such as designated smoking rooms or smoking areas. These policies, align with the WHO FCTC Article 8, ensure 100% smoke-free environments in settings such as restaurants, bars, hotels, and public transportation, protecting people from exposure to SHS.
- Partial Smoke-Free Policy: Policies that allow smoking in certain designated areas or rooms, either indoors or outdoors, while restricting it in others. Such policies permit smoking in specific sections of venues like restaurants or bars, or in designated rooms, but they do not offer full protection from SHS.

We classified economic impacts into four categories: neutral impact, positive impact, negative impact, and mixed impact (indicating both positive and negative impacts).

Following the initial coding process, the articles underwent a second round of review to ensure consistency. The research team reached a consensus on the thematic coding and relevant examples from each article were extracted to illustrate key findings. Insights were then synthesized across the articles to develop a cohesive and comprehensive analysis. A detailed summary of the included studies is available in the Supplementary file, which presents information on the citation, country/jurisdiction, World Bank income classification, WHO region, tourism sector studied, study type, key measures, and the main themes explored in each study. The corresponding citations for each study are also provided in the Supplementary file.

The findings were then reviewed to try and draw connections between the different insights. We also reviewed study designs and analytical methods used in the identified articles to assess the rigor of their analyses. Quasi-experimental designs paired with longitudinal analysis, time series modeling, or generalized estimating equations (GEE) provide a higher degree of rigor in research, particularly when studying interventions or outcomes over time due to their capacity for causal inference and control of confounding factors compared to cross-sectional survey designs^18,19^. In contrast, cross-sectional surveys, qualitative interviews, case studies, and narrative literature reviews offer valuable contextual insights but limited causal or generalizable evidence^18,19^.

**Figure 1:**
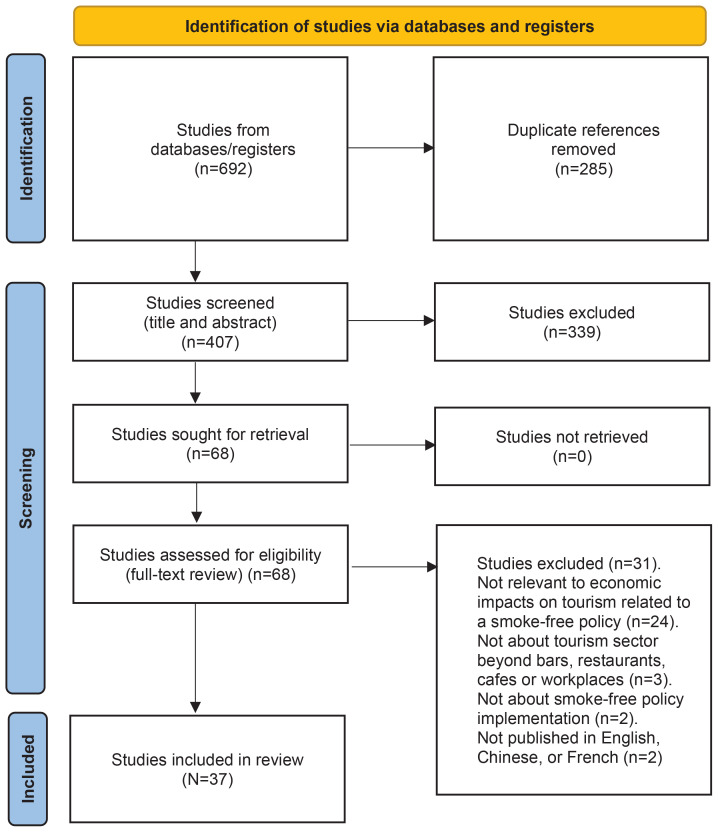
PRISMA flowchart showing the number of citations identified at each stage of the search and screening process

The search produced 692 articles; 285 articles were duplicates and were removed. The title and abstract review identified 407 articles for full text review, which were screened by one coder. This process identified 68 articles that met the inclusion criteria and underwent full-text review by two independent coders. Following full-text review, 31 articles were excluded because they did not present findings relevant to direct or indirect economic impacts on tourism related to a smoke-free policy (24 articles), were not about tourism sector beyond bars/ restaurants/cafes or workplaces (3 articles), were not about smoke-free policy implementation (2 articles), or were not published in English (2 articles). The final sample included in the study was 37 (Figure 1).

### Identifying literature by country and its respective WHO Regional Office and World Bank income classification

The reviewed literature reveals an uneven geographical distribution of studies related to the economic impacts of smoke-free policies on tourism, highlighting an imbalance in research focus. The majority of articles are within the WHO AMRO region, which accounts for 65% (24 studies) of the total. Studies from the United States, Canada, and Caribbean nations dominate the research landscape (Table 1). In contrast, other regions are notably underrepresented, with only 4 studies (10.8%) from the WHO EURO region and 5 studies (14%) from the WPRO region. There is a notable absence of studies from the WHO AFRO, EMRO and SEARO regions, highlighting significant gaps in geographical coverage.

**Table 1:** Number of articles by World Health Organization Regional Office and World Bank Income Classification (N=37)

*Categories*	*% (n)*
**WHO regional office**	
AMRO	64.9 (24)
EURO	10.8 (4)
WPRO	13.5 (5)
Multiple regions	10.8 (4)
**World bank income classification**	
HIC	94.6 (35)
Both LMIC and HIC	5.4 (2)

AMRO: Regional Office for the Americas. EURO: Regional Office for Europe. WPRO: Regional Office for the Western Pacific. HIC: high-income countries. LMIC: low- or middle-income countries.

Furthermore, the vast majority of studies (95%, n=35) focus exclusively on HICs. Only 2 articles (5%) cover both LMICs and HICs, underscoring a substantial research gap concerning the economic impacts of smoke-free policies on tourism in LMICs, especially given the unique economic and cultural contexts in these areas.

### Identifying literature by specific tourism sector

The tourism sectors included in the reviewed articles reflect a focus on a variety of industries impacted by smoke-free policies, with the number of articles discussing tourism sectors beyond restaurants, cafes, and bars summarized in Table 2.

**Table 2:** Number of articles by tourism sector (N=37)

*Tourism sectors*	*% (n)*
Lodging	56.8 (21)
Casinos/gambling	37.8 (14)
Sports	2.7 (1)
Beaches	2.7 (1)
Tourism demand	5.4 (2)

The majority of studies (57%, 21) examine the economic impacts of smoke-free policies on accommodations such as hotels, Airbnb, and motels. These studies explore how smoke-free policies influence guest satisfaction, occupancy rates, and overall revenues in the hospitality industry, reflecting significant interest in how these policies affect guest behavior and business performance. The casino and gambling sector is also heavily represented, with 14 articles (38%) focusing on the impacts of smoke-free policies on this sector. A smaller proportion of articles (8%, 3 articles) examine other tourism sectors, such as beaches and inbound tourism. These studies investigate how smoke-free policies influence tourism patterns, visitor rates, and the overall attractiveness of destinations, providing a broader perspective on how such policies can shape tourism beyond the hospitality and gambling sectors.

### Identifying literature by economic impacts

Of the 37 articles reviewed, the majority (76%) reported neutral or positive economic impacts following the implementation of smoke-free policies (Table 3). Many studies found that hotels, hospitality venues, and some casinos either maintained or improved their revenues after adopting smoke-free environments. For example, McDaniel and Malone ^20^, Alpert et al. ^21^, and Pyles and Hahn^22^ demonstrated that smoke-free policies in hotels and hospitality venues had no significant negative impact on revenues, with some businesses benefiting from increased customer satisfaction and reduced cleaning costs. Noh et al. ^23^ highlighted that smoking bans did not harm revenues in South Korean billiard halls. González-Rozada et al.^24^ found similar results in Caribbean tourism businesses, where smoke-free policies showed no adverse effects on business performance.

**Table 3:** Number of articles by economic impact (N=37)

*Economic impacts*	*% (n)*
**Neutral impact or positive impact**	75.7 (28)
Neutral impact	37.8 (14)
Positive impact	24.3 (9)
Neutral and positive impact	13.5 (5)
Mixed impact*	13.5 (5)
**Negative impact**	10.8 (4)

* The mixed impact includes one of the following combinations: neutral, positive, and negative (n=3); neutral and negative (n=1); or positive and negative (n=1).

Four articles reported negative economic impacts, particularly in sectors heavily dependent on smoking patrons, such as casinos. Lal and Siahpush^25^ found that smoke-free policies led to revenue declines in these sectors, as many patrons sought venues where smoking was still permitted. Brokenleg et al.^26^ noted that casino visitation increased among people who did not smoke. Consequently, revenues may stabilize or even grow as businesses adapt to non-smoking patrons and alternative customer bases.

### Mixed short-term effects with long-term revenue stability from smoke-free policy implementation in tourism

Several studies have demonstrated neutral or positive economic impacts of smoke-free policies on tourism-related revenues, countering concerns about potential financial losses. For example, in a quasi-experimental study in South Korea, Noh et al.^23^ found no negative impact on billiards hall sales following the implementation of smoking bans, using objective credit card sales data to assess revenue. Similarly, Talias et al.^27^ reported no significant reductions in hospitality industry revenues in Cyprus after smoke-free policies were introduced, aligning with findings from Alpert et al.^21^, who evaluated the Massachusetts Smoke-Free Workplace Law and found that the ban’s costs were offset by increased business from people who did not smoke and health-conscious consumers. These studies suggested that while businesses may incur initial adaptation costs, long-term effects tend to be neutral or positive, particularly in settings with substantial non-smoking clients.

However, the economic impact of smoke-free policies on tourism-related sectors can vary, depending on the type of establishment and its patrons. McGrath^28^ found that casinos, especially those catering to people who smoked, experienced short-term revenue declines after smoke-free laws were enacted, as smoking patrons reduced visits or stayed for shorter durations. Lal and Siahpush^25^ found a significant decrease in gaming machine revenue in Victoria, Australia, following smoking bans, as smoking patrons reduced their visits. However, the trend in gambling revenue showed a gradual increase beginning four months after the implementation of the smoke-free policy. Zakarian et al.^29^ similarly reported that some California hotels experienced initial revenue declines during their transition to smoke-free environments, particularly in areas with a high concentration of people who smoked. These examples highlight the adaptation challenges that certain tourism-related businesses may face, with short-term financial strain resulting from the need to attract new, non-smoking clients and implement policy-related changes. In some cases, revenues rebounded as both individuals who smoked and who did not smoke adjusted to the new environment. Tauras et al.^30^ used 18 years of data (10 years before and 8 years after the Illinois law went into effect) and found that although casino admissions decreased in the first quarter after the smoking ban, no significant long-term economic losses were observed after controlling for state-specific, year-specific and quarter-specific determinants of casino activity. The impact of smoke-free policies can be particularly pronounced in tribal casinos, gaming establishments owned and operated by federally recognized Native American tribes on tribal land or reservations, where smoking is often culturally accepted, and policies may differ from state regulations. Nez Henderson et al.^31^ explored the adoption of smoke-free policies on the Navajo Nation and noted concerns about potential revenue losses due to reduced patronage from people who smoked. However, they also suggested that these losses could be mitigated by attracting new health-conscious visitors, leading to more varied long-term impacts depending on how effectively businesses adapt to the new regulations. Overall, while short-term negative impacts are sometimes observed, long-term trends often show recovery or positive economic outcomes as businesses and customers adjust to smoke-free environments.

### Visitor numbers and customer satisfaction: Mixed outcomes with positive long-term trends

Several studies highlight the neutral or positive impacts of smoke-free policies on tourism-related customer behavior, including visitor numbers and customer satisfaction. For instance, González-Rozada et al.^24^ found that comprehensive smoke-free legislation in the Caribbean Community had no negative effect on tourism, with visitor numbers remaining steady or increasing. Similarly, in Hawaii, Dobson Amato et al. ^32^ reported no decline in hotel occupancy or visitor spending five years after the implementation of smoke-free laws. In New Zealand, Brinson et al.^33^ noted strong support from tourists, residents, and businesses for smoke-free and vape-free zones, suggesting that such policies can enhance customer satisfaction without reducing visitor numbers.

However, in certain tourism sectors, such as casinos, the impact of smoke-free policies on visitor numbers and customer satisfaction has been more mixed. Schoen^34^ suggested that while smoking-permitted policies in casinos are often justified by perceived economic benefits, smoking policies may lead to financial losses due to declining customer preference for smoking environments and increased costs related to cleaning, maintenance, and health impacts. In contrast, McGrath^28^ found initial declines in casino admissions as smoking patrons were deterred by the new restrictions. Over time, though, studies like Brokenleg et al.^26^ indicate that customer satisfaction stabilizes, as even smoking patrons adjust to smoke-free environments, continuing to visit due to the overall quality of the venue.

While some sectors may face short-term challenges, particularly in venues that are popular for those who smoke, smoke-free policies can improve the overall visitor experience by enhancing air quality and creating healthier environments. Babb et al.^35^ highlighted the health risks of SHS exposure among casino employees and patrons of casinos that allow smoking. In gaming venues, Klepeis et al.^36^ found that smoke-free policies initially led to reduced admissions and dissatisfaction among people who smoked at a resort/casino. Similarly, Lal and Siahpush^25^ reported declines in monthly gambling revenue, as smoking patrons sought alternative locations. However, these effects are often mitigated over time, as customer preferences shift toward health-conscious environments. Despite initial economic challenges in some sectors, particularly those popular with those that smoke, smoke-free policies generally result in positive long-term outcomes in visitor numbers and customer satisfaction as businesses and patrons adapt.

### Positive employee health outcomes from smoke-free policy implementation in tourism

Smoke-free policies have demonstrated significant positive impacts on employee health outcomes, particularly in terms of reducing absenteeism and healthcare costs. Rajkumar et al.^37^ found that workplace smoking bans led to measurable improvements in cardiovascular health among hospitality workers, which translated into reduced health care expenses. Similarly, Alpert et al.^21^ reported that the Massachusetts Smoke-Free Workplace Law improved air quality for employees and contributed to lower healthcare costs, highlighting the broader benefits of smoke-free environments in the workplace. These improvements not only enhance the well-being of workers but also boost overall productivity by reducing absenteeism and turnover rates.

In terms of health, the evidence is overwhelmingly positive. Siegel et al. ^38^ showed that hospitality workers covered by smoke-free laws experienced fewer respiratory issues and overall better health outcomes due to reduced exposure to SHS. This resulted in long-term cost savings for employers, as healthier employees required fewer sick days and medical treatments. Eisner^39^ further emphasized the positive health impacts of smoke-free policies for hospitality workers, reinforcing that such policies reduce SHS exposure, which decreases absenteeism and improves long-term health outcomes. However, the employment impact of smoke-free policies presents more complex consequences. In some cases, such as in casinos or hotels experiencing temporary revenue declines post-policy, businesses may reduce staff hours or even resort to layoffs as they adjust to the customer base that prefers a smoke-free environment^28,^ ^31^. This demonstrates a potential tradeoff between improved employee health and short-term disruptions in employment stability.

### Comprehensive versus partial smoke-free policies: Impact on tourism and enforcement challenges

The effectiveness of smoke-free policies is closely related to whether they are comprehensive or partial. Comprehensive smoke-free policies, which prohibit smoking in all indoor public spaces without any exceptions, have been consistently shown to yield more positive outcomes for both public health and the economy. For instance, González-Rozada et al.^24^ found that comprehensive smoke-free legislation in the Caribbean did not negatively impact tourism. The consistent enforcement of these policies contributed to healthier environments for both customers and employees, enhancing visitor satisfaction in some cases. Similarly, Alpert et al.^21^ and Talias et al.^27^ suggested that comprehensive policies create a clearer framework for businesses, fostering a positive image for the tourism sector and attracting health-conscious visitors.

In contrast, partial smoke-free policies, which allow smoking in designated areas or rooms, pose enforcement challenges and reduce overall health benefits. Nez Henderson et al.^31^ argued that partial bans may undermine the effectiveness of smoke-free environments, as they do not fully protect employees and non-smoking patrons. Klepeis et al.^36^ also noted that while air quality improved at a resort/casino after the implementation of partial smoking bans, the casino faced resistance from smoking patrons, which raised concerns about revenue losses.

From an economic perspective, the mixed impacts of smoke-free policies are often influenced by whether the policies are comprehensive or partial. Comprehensive bans, though occasionally facing resistance from businesses that cater to people who smoke, tend to offer clearer long-term benefits. Tauras et al.^30^ found that the smoking bans initially led to revenue declines in casinos due to their high proportion of smoking patrons, but these impacts stabilized over time as businesses adapted. On the other hand, partial bans or exemptions, such as allowing smoking in certain sections of casinos or hotels, tend to result in negative economic outcomes. Zakarian et al.^29^ highlighted that hotel managers in California faced initial declines in guest numbers and dissatisfaction from smoking patrons following the adoption of smoke-free policies. Similarly, Lal and Siahpush^25^ demonstrated that partial smoking bans in gaming venues in Victoria, Australia, contributed to declines in gaming revenue, as smoking patrons frequented venues less often or sought alternatives where smoking was allowed. These findings suggest that while partial policies may reduce immediate economic shocks by allowing smoking in certain areas, they also limit the potential health benefits and may result in long-term mixed or negative economic outcomes, especially in venues with a high concentration of smoking patrons. Also, venues with partial exemptions create inconsistencies in customer experience, fail to fully address health concerns, and complicate enforcement that may make them less attractive to new customers.

### Varying economic impacts of smoke-free policies across tourism sectors: Positive outcomes in hotels, mixed results in casinos

The economic impacts of smoke-free policies vary significantly across different tourism sectors, with studies on hotels, motels, and Airbnb accommodations consistently showing neutral or positive outcomes. For example, John et al.^40^ found that smoke-free laws in South Australia’s hotels and licensed clubs had no negative impact on revenues. Similarly, McDaniel and Malone^20^ reported that hoteliers who implemented 100% smoke-free guest-room policies experienced high levels of satisfaction from customers and employees and improvement in operational efficiency by not having to balance between smoking and non-smoking rooms, suggesting that smoke-free policies may even enhance business performance by attracting health-conscious travelers and families and create a healthier working environment. In the hotel industry, smoke-free environments are frequently associated with improved air quality and increased occupancy rates over time, despite initial concerns about alienating smoking patrons. Christophi et al.^41^ found that hotels with smoke-free policies saw improvements in hotel turnover rate – measured as the number of guests staying at the hotel over a given period – reflecting increased occupancy and customer flow (a 4.1% increase), and a 7.2% increase in employment during the year of policy implementation.

While some tourism sectors, particularly casinos, may experience short-term economic disruptions after adopting smoke-free policies, the long-term benefits in terms of customer satisfaction and employee health often outweigh these initial challenges. Studies by Tauras et al.^30^ found no significant long-term decline in casino admissions or revenues after the implementation of the Smoke-Free Illinois Act. Although casinos, which cater to a higher proportion of smoking patrons, may experience short-term losses due to reduced patronage by those that smoke, many businesses eventually adapt, and revenues stabilize as non-smoking customers replace individuals who smoke. Negative impacts in casinos are more pronounced in the short-term, as seen in the studies by Klepeis et al. ^36^ and Lal and Siahpush^25^, where revenues initially declined after smoke-free policies were implemented. However, Lal and Siahpush^25^ suggested that casinos may recover over time as they attract new patrons or adjust their offerings to meet changing customer preferences. Even in traditionally smoking-heavy environments like tribal casinos, Schoen and Brokenleg et al.^34^ found that offering non-smoking amenities helped maintain patronage levels despite initial pushback from smoking patrons. The casino sector faces more immediate challenges. However, even in casinos, revenues often stabilize as businesses adapt to smoke-free environments, highlighting the potential for long-term recovery despite initial declines.

### Assessing methodological rigor and bias

Among the 37 studies we reviewed, approximately one-third (n=13) employed rigorous study designs capable of supporting causal inference and controlling for confounding factors (Supplementary file). Studies reporting negative economic impacts of smoke-free policies often rely on methodologies that lack robustness, undermining the validity of their conclusions. For example, Zakarian et al.^29^ surveyed hotel managers in California about the transition to smoke-free environments but based their findings primarily on subjective self-reports without corroborating the claims with objective financial data or broader market trends. The study also fails to control for confounding variables, such as regional economic trends or shifts in the tourism market, making it difficult to attribute any observed outcomes directly to the implementation of smoke-free policies. Moreover, the absence of comparison groups or control mechanisms limits the study’s ability to draw causal conclusions about the economic impacts of these policies. Similarly, Klepeis et al.^36^ conducted an air quality study at a resort/casino, reporting qualitative insights from stakeholders regarding the economic impact of partial smoke-free policies. However, the reliance on anecdotal evidence and the lack of concrete financial data reduce the generalizability of the findings. In Sherlock^42^, the analysis of the Iowa Smokefree Air Act’s gaming-floor exemption provides a thorough legal review, but its discussion of economic impacts is largely speculative and lacks empirical data to support the claims. The study does not adequately address other factors, such as changes in local tourism or broader economic conditions, leaving its conclusions vulnerable to bias and overgeneralization. Without a rigorous quantitative assessment, the study offers limited insight into the actual economic consequences of partial smoke-free policies on casino revenues and patronage. Lal and Siahpush^25^ offer a quantitative examination of the impact of smoke-free policies on electronic gaming machine expenditure in Victoria, Australia. However, the study’s short time frame following the policy implementation only captures initial revenue declines while failing to account for potential long-term trends. Additionally, the absence of control for external factors, such as changes in gaming behavior or broader economic fluctuations, weakens the study’s ability to draw robust conclusions about the sustained economic impact of smoke-free policies.

Further complicating the reliability of studies reporting negative impacts is the influence of the tobacco industry. Siegel et al.^38^ and Scollo et al.^15^ reviewed research funded by the tobacco industry and found that such research often overemphasizes negative economic impacts by using selective data and methodologies designed to amplify economic harm. This bias is driven by the industry’s vested interest in maintaining smoking-friendly environments, raising concerns about the credibility of these findings.

In contrast, research with more rigorous methodologies consistently finds that any negative impacts from smoke-free policies are short-term and often outweighed by long-term benefits. For instance, several studies conducted time series analyses to explore the influence of smoke-free policy on tourism, concluding that these policies had a neutral or positive economic impact conducted a longitudinal study to examine tourist arrivals and monthly spending using linear regression that controlled for seasonal and economic trends^22,27,40,43^. They found no evidence of harm to Hawaii’s tourism and hospitality industries five years after implementing smoke-free laws. McMillen and Shackelford^44^ conducted multivariate fixed-effects analyses to examine the change in tax revenue before and after the implementation of smoke-free policy. The results suggested that there was no economic impact on tourism-related tax revenues. These findings suggest that initial revenue losses are typically offset by long-term gains, including healthier environments for employees and customers, ultimately leading to more sustainable business models. This contrast between rigorous, independent studies and those influenced by industry bias, underscores the importance of methodological rigor in assessing the true economic impacts of smoke-free policies on tourism.

The evidence reviewed in this study indicates that smoke-free policies generally have neutral or positive economic impacts on tourism, including lodging, casinos/gambling, sports, and beaches, beyond restaurants, bars and cafes. Studies show that smoking bans may initially reduce revenues in some businesses, notably casinos, where a significant portion of patrons smoke. However, these revenue declines are typically temporary, and long-term benefits tend to emerge as businesses and customers adjust to the new smoke-free environments. The findings suggest that comprehensive smoke-free policies are more effective at promoting public health and lowering government spending on healthcare while maintaining or improving economic performance in tourism-related sectors. In contrast, partial bans offer mixed results, often complicating enforcement and limiting the health benefits of the policies.

The hotel sector has been particularly successful in adapting to smoke-free policies, with studies reporting improvements in guest satisfaction and stable occupancy rates. Hotels have seen long-term benefits, including better air quality and a greater appeal to non-smoking and health-conscious guests. On the other hand, casinos, which traditionally cater to a higher proportion of patrons who smoke, tend to face more immediate economic challenges post-ban. However, these businesses typically recover over time, as seen in several studies, and revenue levels stabilize once both smoking and non-smoking patrons adjust to the new regulations. McGrath^28^ conducted a literature review of the impact of smoking bans on revenues from casinos/gambling venues, finding mixed results, with some studies showing little effect on casino revenues, while some indicated significant declines within one year of smoke-free policy implementation. They emphasized the need for longitudinal research to provide more robust evidence on the economic effects of smoke-free policies in the gambling sector. There is a lack of literature examining sectors beyond hotels and casinos. For other sectors, such as entertainment venues, the available evidence generally suggests positive outcomes, as smoke-free environments attract a broader range of visitors and enhance overall customer experience and thus satisfaction.

In addition, it is important to note the potential differences between mandated national or state-level smoke-free policies and voluntary policies implemented by individual businesses or chains. When all venues of a particular sector (e.g. casinos, hotels) simultaneously implement smoke-free policies, economic and behavioral adjustments may occur more rapidly and uniformly across the sector. However, the studies reviewed do not allow for direct observation of the effects of such simultaneous policy implementation. Pyles and Hahn^43^ similarly reviewed health and economic outcomes of smoke-free legislation, emphasizing the importance of effective implementation to achieve the intended benefits, particularly among vulnerable subpopulations.

A key gap in the literature is the lack of studies on smoke-free policies in LMICs, particularly in the WHO African and South-East Asia Regions. Most research to date has focused on HICs such as the United States, Canada, and European nations, with only limited attention given to regions like the Caribbean and Cyprus. This regional bias limits the generalizability of findings, as tourism sectors in LMICs face unique economic, social, and regulatory challenges that could influence the outcomes of smoke-free policy implementation. The absence of research in LMICs, especially in the WHO AFRO and SEARO, also hampers understanding of how these policies affect tourism in regions with high tobacco use prevalence and developing tourism industries. Addressing this gap is essential for enabling evidence-based policymaking and ensuring that the health and economic benefits of smoke-free policies in tourism are fully realized across diverse global contexts. By expanding research beyond high-income countries and including diverse tourism sectors, policymakers can make informed decisions that account for regional and economic differences, thereby optimizing both public health outcomes and the sustainability of tourism industries worldwide.

### Strengths and limitations

This narrative review provides a comprehensive overview of the economic impacts of smoke-free policy implementation across a range of tourism sectors, including hotels, casinos, and other hospitality venues. A key strength lies in the diversity of the evidence included, covering neutral, positive and negative economic impacts, as well as different types of smoke-free policies (comprehensive vs partial). This diversity allows for a nuanced understanding of how smoke-free policies affect various sectors, contributing to a well-rounded perspective. The inclusion of both academic sources and gray literature (such as government reports and industry assessments) further enhances the breadth of analysis, as it incorporates real-world insights that are often missed in purely peer-reviewed studies. This mixed-source approach provides a holistic view of the practical implications of smoke-free policies, capturing emerging trends and allowing for a more grounded understanding of their economic effects. Additionally, the review’s focus on multiple countries and sectors, strengthens the analysis by enabling comparison of economic outcomes across diverse countries and tourism industries. Furthermore, this review followed the principles of a narrative review, which differs from more systematic approaches in several ways. The study excluded steps typically taken in systematic reviews, such as conducting a risk of bias assessment or using a PICO framework for data extraction^45^. While this approach allowed for a broad overview of global evidence on smoke-free policies, it inherently sacrifices some rigor in methodological appraisal. However, the narrative review design enabled the research team to answer a research question with broader parameters, providing a nuanced understanding of the complexities surrounding economic impacts of smoke-free policies, which may not have been possible within the confines of a more structured review.

Despite these strengths, one limitation should be acknowledged: the language restriction, as only articles published in English were included. This language barrier likely excludes relevant studies published in other languages, particularly those from LMICs and the WHO African and South-East Asia Regions, where the economic impacts of smoke-free policies on tourism may differ significantly from those observed in HICs and other WHO regions. The language restriction narrows the scope of the review and may overlook valuable data from regions where tourism is rapidly growing but underrepresented in the reviewed languages. Another limitation is that, due to the qualitative nature of our synthesis and the diversity in study designs, populations, and outcomes, a formal quantitative analysis or meta-analysis could not be performed. Furthermore, the considerable heterogeneity among the included studies, in terms of methodology, sample size, measured variables, and reporting, restricts our ability to directly compare findings across studies.

## CONCLUSION

While the economic impacts of smoke-free policy implementation on tourism can vary by sector, the overall evidence strongly supports their long-term benefits, particularly when implemented comprehensively. The health improvements for employees and visitors, combined with sustained or even improved revenue streams in many sectors, highlight the value of smoke-free policies in promoting both public health and economic sustainability within the tourism industry. Expanding research into LMICs is essential to fully understand the global implications of the economic impacts of smoke-free policies and to ensure that the tourism industry can benefit from the economic and health advantages of these measures across diverse regions.

## Supplementary Material



## Data Availability

The data supporting this research are available from the authors on reasonable request.
