# Supplementary material for: State of the evidence on economic impacts of smoke-free policies in the tourism sector: A narrative literature review
Source: Tob Induc Dis. 2025 Dec 4;23:10.18332/tid/211072. doi: 10.18332/tid/211072 (PMC12677000; doi:10.18332/tid/211072)
Supplement: Supplementary file 1 [file TID-23-189-s1.pdf]

Supplementary Table 1: A summary of the included studies

Citation	Country/ Jurisdiction	World Bank income classification	WHO region	Tourism sector	Study type	Key measures	Main themes explored
Selin H. Tourism and smoke-free environments: the evidence. *Tour Hosp Plan Dev.* 2005;2(1):61-63. https://doi.org/10.1080/14790530500072286	USA	HIC	AMRO	Lodging	Review	Sales tax, employment	Examines the economic impact of smoke-free environments on tourism and hospitality sectors
Siegel M, Barbeau EM, Osinubi OY. The impact of tobacco use and secondhand smoke on hospitality workers. *Clin Occup Environ Med.* 2006;5(1):31-42, viii. https://doi.org/10.1016/j.coem.2005.10.005	USA	HIC	AMRO	Lodging	Review	Revenue, employment	Explores the health and economic impact of tobacco use and secondhand smoke on hospitality workers
Alpert HR, Carpenter CM, Travers MJ, Connolly GN. Environmental and economic evaluation of the Massachusetts smoke-free workplace law. *J Community Health.* 2007;32(4):269-281. https://doi.org/10.1007/s10900-007-9048-6	Massachusetts, USA	HIC	AMRO	Lodging	Evaluation	Sales, tax, employment	Assesses the economic and environmental impact of the smoke-free law

Citation	Country/ Jurisdiction	World Bank income classification	WHO region	Tourism sector	Study type	Key measures	Main themes explored
Allwright S. The impact of banning smoking in workplaces. *Appl Health Econ Health Policy*. 2008;6(2-3):81-92. https://doi.org/10.1007/bf03256124	Ireland, Scotland, other HICs	HIC	AMRO, EURO, WPRO	Lodging	Review	Revenue, maintenance cost, absenteeism	Reviews early impacts of smoke-free workplace laws globally
Edwards R, Thomson G, Wilson N, et al. After the smoke has cleared: evaluation of the impact of a new national smoke-free law in New Zealand. *Tob Control*. 2008;17(1):e2-e2. https://doi.org/10.1136/tc.2007.020347	New Zealand	HIC	WPRO	Lodging, casinos/gambling, and tourism	Survey, Evaluation	Health outcomes, retail sales, number of overseas visitors, tourism expenditure	Assesses economic, health, and compliance impacts of New Zealand's smoke-free law
Zelnick J, Campbell R, Levenstein C, Balbach E. Clearing the air: the evolution of organized labor's role in tobacco control in the United States. *Int J Health Serv*. 2008;38(2):313-331. https://doi.org/10.2190/HS.38.2.f	USA	HIC	AMRO	Lodging	Case study, Historical analysis	Employment, health outcomes	Examines the U.S. labor movement's evolving role in tobacco control

Citation	Country/ Jurisdiction	World Bank income classification	WHO region	Tourism sector	Study type	Key measures	Main themes explored
Samet JM. Secondhand smoke: facts and lies. *Salud Publica Mex*. 2008;50(5):428-434. https://doi.org/10.1590/s0036-36342008000500016	Mexico	LMIC	AMRO	Lodging, casinos/gambling	Review	Revenue, health outcomes	Reviews economic implications of secondhand smoke and smoke-free policies on the hospitality industry
Lal A, Siahpush M. The effect of smoke-free policies on electronic gaming machine expenditure in Victoria, Australia. *J Epidemiol Community Health*. 2008;62(1):11-15. https://doi.org/10.1136/jech.2006.051557	Victoria, Australia	HIC	WPRO	Casinos/Gambling	Interrupted time-series*	Electronic gaming machine expenditure (the smoke-free policy associated with a 14% decrease in the monthly expenditure, $p<0.05$)	Analyzes the impact of smoke-free policy on gambling expenditure
Sherlock K. Clearing the air: analyzing the constitutionality of the Iowa smoke-free Air Act's gaming-floor exemption. *Iowa Law Rev*. 2009;95:347-388.	USA	HIC	AMRO	Casinos/Gambling	Review	Revenue	Legal and economic analysis of smoke-free policies with gambling exemptions
Eisner MD. Secondhand smoke exposure and the	USA, Ireland,	HIC	AMRO/	Lodging, casinos/gam	Review / Book	Health outcomes,	Reviews secondhand smoke exposure risks

Citation	Country/ Jurisdiction	World Bank income classification	WHO region	Tourism sector	Study type	Key measures	Main themes explored
health of hospitality workers. In: Tarlo SM, Cullinan P, Nemery B, eds. *Occupational and Environmental Lung Diseases*. Wiley-Blackwell; 2010:121-128. https://doi.org/10.1002/9780470710425.ch9	Scotland, Norway		EURO	bling	Chapter	revenue	for hospitality workers
Hahn EJ. smoke-free legislation: a review of health and economic outcomes research. *Am J Prev Med*. 2010;39(6 Suppl 1):S66-S76. https://doi.org/10.1016/j.amepre.2010.08.013	USA	HIC	AMRO	Lodging	Review	Retail sales tax, employment, revenue, operating cost	Review of smoke-free legislation impacts on business revenue, operating costs and employment
Harris JK, Carothers BJ, Luke DA, Silmere H, McBride TD, Pion M. Exempting casinos from the Smoke-Free Illinois Act will not bring patrons back: they never left. *Tob Control*. 2012;21(3):373-376. https://doi.org/10.1136/tc.2010.042127	Illinois, USA (compared with Indiana, Iowa, Missouri)	HIC	AMRO	Casinos/ Gambling	Pre-post quasi-experimental*	Casino admission (no significant changes after the implementation of smoke-free policy, p>0.05)	Evaluates the impact of Illinois' Smoke-Free Act on casino admissions
John DL, Bowden JA, Miller CL. The impact of smoke-free	South	HIC	WPRO	Lodging	Time-series data	Monthly retail turnover (no	Analyzes the economic impact of

Citation	Country/ Jurisdiction	World Bank income classification	WHO region	Tourism sector	Study type	Key measures	Main themes explored
laws on business revenue in hotels and licensed clubs in South Australia. *Aust N Z J Public Health*. 2011;35(3):295-296. https://doi.org/10.1111/j.1753-6405.2011.00709.x	Australia				analysis (ARIMA models)*	significant association with the smoke-free laws, p>0.05)	smoke-free laws
Pyles MK, Hahn EJ. Economic effects of smoke-free laws on rural and urban counties in Kentucky and Ohio. *Nicotine Tob Res*. 2012;14(1):111-115. https://doi.org/10.1093/ntr/ntr123	Kentucky/Ohio border counties, USA	HIC	AMRO	Lodging	Time-series analysis (FLGS model)*	Employment, wages paid, number of establishments (no significant relation between the smoking ban and the economic indicator variables, p>0.05)	Evaluates the economic impact of Ohio's smoke-free law on border counties
Timberlake DS, Wu J, Al-Delaimy W. Tribal casinos in California: the last vestige of indoor smoking. *BMC Public Health*. 2012;12(1):144. https://doi.org/10.1186/1471	California, USA	HIC	AMRO	Casino/ Gambling	Cross-sectional survey	Casino admission	Explores the impact of hypothetical comprehensive smoke-free policies in tribal casinos

Citation	Country/ Jurisdiction	World Bank income classification	WHO region	Tourism sector	Study type	Key measures	Main themes explored
-2458-12-144							
McMillen R, Shackelford S. Tax revenue in Mississippi communities following implementation of smoke-free ordinances: an examination of tourism and economic development tax revenues. *J Miss State Med Assoc*. 2012;53(10):319-321	Mississippi, USA	HIC	AMRO	Lodging	Pre-post quasi-experimental*	Tax revenue (no significant effect of smoke-free policies on hospitality tax revenue, p>0.05)	Examines the impact of smoke-free ordinances on tourism and economic development
Pyles MK, Hahn EJ. Economic effects of Ohio's smoke-free law on Kentucky and Ohio border counties. *Tob Control*. 2011;20(1):73-76. https://doi.org/10.1136/tc.2009.035493	Kentucky and Ohio, USA	HIC	AMRO	Lodging	Generalized estimating equation (GEE) time-series study*	Employment, wages paid, number of establishments (no significant relation between the smoking ban and the economic indicator variables, p>0.05)	Assesses economic impact of smoke-free laws

Citation	Country/ Jurisdiction	World Bank income classification	WHO region	Tourism sector	Study type	Key measures	Main themes explored
Ariza E, Leatherman SP. No-smoking policies and their outcomes on U.S. beaches. *J Coast Res*. 2012;27(8):143-147. https://doi.org/10.2112/JCOASTRES-D-10-00137.1	USA	HIC	AMRO	Beaches	Case study	Perception of tourism appeal	Examines the outcomes of no-smoking policies on U.S. beaches
Christophi CA, Paisi M, Pampaka D, Kehagias M, Vardavas C, Connolly GN. The impact of the Cyprus comprehensive smoking ban on air quality and economic business of hospitality venues. *BMC Public Health*. 2013;13(1):76. https://doi.org/10.1186/1471-2458-13-76	Cyprus	HIC	EURO	Lodging	Pre-post quasi-experimental*	Revenue, employment (the hotel turnover rate increased by 4.1% and employment increased by 7.2% after implementation of the smoking ban)	Assesses the economic and air quality impacts of Cyprus' comprehensive smoking ban
Dobson Amato KA, Rivard C, Lipsher J, Hyland A. Five years after the Hawai'i smoke-free law: tourism and hospitality economic indicators appear unharmed. *Hawai'i J Med Public Health*.	Hawaii, USA	HIC	AMRO	Tourism demand	Longitudinal study*	Visitor arrivals, employment (the smoke-free policy significantly associated with an increase in arrivals [β=	Evaluates the impact of Hawaii's smoke-free law over five years

Citation	Country/ Jurisdiction	World Bank income classification	WHO region	Tourism sector	Study type	Key measures	Main themes explored
2013;72(10):355-361.						42847.9; 95% CI: 16303.3, 69392.5], and employees [β=3390.8; 95% CI: 2326.9, 4454.7])	
Babb S, McNeil C, Kruger J, Tynan MA. Secondhand smoke and smoking restrictions in casinos: a review of the evidence. *Tob Control*. 2015;24(1):11-17. https://doi.org/10.1136/tobaccocontrol-2013-051368	USA, Australia (Victoria)	HIC	AMRO, WPRO	Casinos/ Gambling	Literature Review	Air quality, SHS exposure biomarkers, health outcomes, smoking prevalence	Reviews economic and health impacts of smoke-free policies in casinos
Brokenleg I, Barber TK, Bennett NL, Peart Boyce S, Blue Bird Jernigan V. Gambling with our health: smoke-free policy would not reduce tribal casino patronage. *Am J Prev Med*. 2014;47(3):290-299. https://doi.org/10.1016/j.amepre.2014.04.006	USA	HIC	AMRO	Casinos/ Gambling	Survey	Casino admission	Evaluates the potential impact of smoke-free policies in tribal casinos

Citation	Country/ Jurisdiction	World Bank income classification	WHO region	Tourism sector	Study type	Key measures	Main themes explored
Rajkumar S, Schmidt-Trucksäss A, Wellenius GA, et al. The effect of workplace smoking bans on heart rate variability and pulse wave velocity of non-smoking hospitality workers. *Int J Public Health*. 2014;59(4):577-585. https://doi.org/10.1007/s00038-014-0545-y	Switzerland	HIC	EURO	Lodging	Quasi-experimental*	Cardiovascular risk indicators (PWV [p<0.001] and HRV [p=0.02] parameters significantly improved after the implementation of smoke-free policy)	Examines the impact of Switzerland's partial smoke-free policy on non-smoking hospitality workers' health
McGrath DS. The influence of smoke-free policies on gambling revenues and intentions to gamble: a review of the literature. *Can J Addict*. 2015;6(2):78-86. https://doi.org/10.1097/02024458-201509000-00011	Canada, U.S. (Delaware, Illinois, Indiana, California), Australia, Ireland	HIC	AMRO, WPRO, EURO	Casinos/ Gambling	Literature review	Revenue, intentions to gamble	Reviews global evidence on the economic impact of smoke-free policies on gambling venues
Talias MA, Savva CS, Soteriades ES, Lazuras L. The effect of smoke-free policies on hospitality industry revenues in Cyprus: an econometric approach. *Tob*	Cyprus	HIC	EURO	Lodging	Time-series analysis*	Revenue adjusted for GDP, inflation, unemployment, tourist arrivals (no	Uses econometric analysis to assess smoke-free policy impacts

Citation	Country/ Jurisdiction	World Bank income classification	WHO region	Tourism sector	Study type	Key measures	Main themes explored
Control. 2015;24(e3):e199-e204. https://doi.org/10.1136/tobaccocontrol-2013-051477						significant effect of the implementation of smoke-free policy on these measures, p>0.05)	
Nez Henderson P, Roeseler A, Moor G, et al. Advancing smoke-free policy adoption on the Navajo Nation. *Tob Control*. 2016;25(Suppl 1):i26-i31. https://doi.org/10.1136/tobaccocontrol-2016-053109	Navajo Nation (New Mexico, Arizona, Utah), USA	HIC	AMRO	Casinos/ Gambling	Review	Revenue, employment, health outcomes	Discusses the political and economic debate over smoke-free policy adoption on the Navajo Nation
Chan ESW, Hsu CHC. Environmental management research in hospitality. *Int J Contemp Hosp Manag*. 2016;28(5):886-923. https://doi.org/10.1108/IJCHM-02-2015-0076	Global	Not Applicable	Not Applicable	Lodging	Literature review	Indoor air quality, cost	Reviews the evolution of environmental management research in hospitality
Klepeis N, Dhaliwal N, Hayward G, et al. Measuring indoor air quality and	California, USA	HIC	AMRO	Casinos/ Gambling	Survey, Focus Groups,	Revenue, perspectives	Documents the implementation challenges of a 100%

Citation	Country/ Jurisdictio n	World Bank income classificatio n	WHO region	Tourism sector	Study type	Key measures	Main themes explored
engaging California Indian stakeholders at the Win-River Resort and Casino: collaborative smoke-free policy development. *Int J Environ Res Public Health*. 2016;13(1):143. https://doi.org/10.3390/ijerph13010143					Interviews, Air Quality Monitoring	of stakeholder	smoke-free policy at a tribal casino
Zakarian JM, Quintana PJE, Winston CH, Matt GE. Hotel smoking policies and their implementation: a survey of California hotel managers. *Tob Induc Dis*. 2017;15(1):40. https://doi.org/10.1186/s12971-017-0147-6	California, USA	HIC	AMRO	Lodging	Survey	Maintenance costs	Examines the economic and operational impact of hotel smoking policies in California
Kennedy RD, Douglas O, Stehouwer L, Dawson J. The availability of smoking-permitted accommodations from Airbnb in 12 Canadian cities. *Tob Control*. 2018;27(1):112-116. https://doi.org/10.1136/tobaccocontrol-2016-053315	Canada	HIC	AMRO	Lodging	Cross-sectional	Room price	Analyzes the availability and pricing of smoking-permitted vs. smoke-free Airbnb accommodations

Citation	Country/ Jurisdiction	World Bank income classification	WHO region	Tourism sector	Study type	Key measures	Main themes explored
McDaniel PA, Malone RE. “You want your guests to be happy in this business”: hoteliers’ decisions to adopt voluntary smoke-free guest-room policies. *Am J Health Promot*. 2018;32(8):1740-1746. https://doi.org/10.1177/0890117118763742	San Francisco, Las Vegas, Miami Beach, USA	HIC	AMRO	Lodging	Interviews	Guest requests, cost	Explores hotel decision-making around voluntary smoke-free guest-room policies
Tauras JA, Chaloupka FJ, Moor G, Henderson PN, Leischow SJ. Effect of the Smoke-Free Illinois Act on casino admissions and revenue. *Tob Control*. 2018;27:e130-e135. https://doi.org/10.1136/tobaccocontrol-2017-053966	Illinois, USA (compared with Indiana, Iowa, Missouri)	HIC	AMRO	Casinos / Gambling	Quasi-experimental*	Casino admission, revenue (no significant effect of the implementation of smoke-free policy on these measures, p>0.05)	Analyzes the economic impact of the Smoke-Free Illinois Act on casino admissions and revenue
González-Rozada M, Prieto-Lara E, Sandoval GA. Effect of comprehensive smoke-free legislation on the tourism industry in countries of the Caribbean Community. *Rev*	Barbados, Guyana, Jamaica, Trinidad and Tobago	HIC/LMIC	AMRO	Tourism demand	Quasi-experimental*	Tourist arrivals, expenditure, average length of stay (no significant	Examines the economic impact of comprehensive smoke-free policies on the Caribbean

Citation	Country/ Jurisdiction	World Bank income classification	WHO region	Tourism sector	Study type	Key measures	Main themes explored
Panam Salud Publica. 2022;46:e146. https://doi.org/10.26633/RPSP.2022.146						change in these measures after the implementation of smoke-free policy, p>0.05)	tourism industry
Brinson D, Ward C, Ford C, Begg A. Smoke-free and vapefree streets: high levels of support from tourists, residents and businesses, implications for tourist-destination communities in New Zealand. *New Zealand Medical Journal*. 2022;135(1559):73-84. https://doi.org/10.26635/6965.5202	Hanmer Springs, New Zealand	HIC	WPRO	Lodging	Evaluation	Visitor counts	Evaluates the impact of a smoke-free/vapefree street trial in a tourist town; finds high support from tourists, residents, and businesses
Noh, J., Cheon, J., Seong, H., Kwon, Y. D., & Yoo, K. (2024). Impacts of Smoking Ban Policies on Billiard Hall Sales in South Korea Using	South Korea	HIC	WPRO	Sports	Quasi-experimental*	Sales (no significant change after the smoking	Assesses the economic impact of South Korea's indoor sports facility smoking

Citation	Country/ Jurisdiction	World Bank income classification	WHO region	Tourism sector	Study type	Key measures	Main themes explored
Objective Sales Information of a Credit Card Company: Quasi-Experimental Study. *JMIR Public Health and Surveillance*, 10, e50466. https://doi.org/10.2196/50466						ban, p>0.05)	ban on billiard halls

Notes: *Rigorous study design with the capacity for causal inference and control for confounding factors.

© 2025 Nian Q. et al.
